# The use of routine blood tests to assist the diagnosis of COVID-19 in symptomatic hospitalized patients

**DOI:** 10.1177/0004563221999076

**Published:** 2021-03-09

**Authors:** IT Parsons, AT Parsons, E Balme, G Hazell, R Gifford, M Stacey, DR Woods, D Russell-Jones

**Affiliations:** 1 School of Life Sciences and Medicine, King’s College London, St Thomas’ Hospital, London, UK; 2 School of Electronic, Electrical & Systems Engineering, Edgbaston, University of Birmingham, Birmingham, UK; 3 Royal Surrey County Hospital NHS Trust, Guildford, UK; 4 Academic Department of Military Medicine, Research and Clinical Innovation, Royal Centre for Defence Medicine, Birmingham, UK

**Keywords:** Haemoglobin, proteins, troponin

## Abstract

**Introduction:**

Specific patterns of blood test results are associated with COVID-19 infection. The aim of this study was to identify which blood tests could be used to assist in diagnosing COVID-19.

**Method:**

A retrospective review was performed on consecutive patients referred to hospital with a clinical suspicion of COVID-19 over a period of four weeks. The patient’s clinical presentation and severe acute respiratory syndrome coronavirus 2 reverse-transcription polymerase chain reaction (SARS-CoV-2 RT-PCR) were recorded. The patients were divided by diagnosis into COVID (COVID-19 infection) or CONTROL (an alternate diagnosis). A retrospective review of consecutive patients over a further two-week period was used for the purposes of validation.

**Results:**

Overall, 399 patients (53% COVID, 47% CONTROL) were analysed. White cell count, neutrophils and lymphocytes were significantly lower, while lactate dehydrogenase and ferritin were significantly higher, in the COVID group in comparison to CONTROL. Combining the white cell count, lymphocytes and ferritin results into a COVID Combined Blood Test (CCBT) had an area under the curve of 0.79. Using a threshold CCBT of –0.8 resulted in a sensitivity of 0.85 and a specificity of 0.63. Analysing this against a further retrospective review of 181 suspected COVID-19 patients, using the same CCBT threshold, resulted in a sensitivity of 0.73 and a specificity of 0.75. The sensitivity was comparable to the SARS-CoV-2 RT PCR.

**Discussion:**

Mathematically combining the blood tests has the potential to assist clinical acumen allowing for rapid streaming and more accurate patient flow pending definitive diagnosis. This may be of particular use in low-resource settings.

## Introduction

The diagnosis of COVID-19, particularly in those not requiring respiratory support, hinges on the detection of severe acute respiratory syndrome coronavirus 2 (SARS-CoV-2) RNA by reverse-transcription polymerase chain reaction (RT-PCR) following sampling of the upper respiratory tract via a nasopharyngeal swab. While a positive test for SARS-CoV-2 generally confirms the diagnosis of COVID-19, false-negative tests, from upper respiratory tract sampling, have been well documented^1^ and could be as high as 20%.^2–4^ False negatives can be due to the specific RT-PCR assay, poor sampling technique, the type and quality of the specimen obtained and the duration of the illness prior to testing. There are significant consequences of missing diagnoses such as propagating viral transmission. This is particularly important in the occupational management of key workers as well as non-infected patients.

It has been established that COVID-19 infection is associated with patterns of blood test results among hospitalized patients. These include a raised lactate dehydrogenase (LDH), inflammatory markers (e.g. ferritin [FER], C-reactive protein [CRP], aminotransferases and d-dimers^5,6^) and lymphopenia. These blood tests, at particular levels, have been associated with a worse prognosis in COVID-19 patients^7–9^ and have been shown to differentiate between those who are SARS-CoV-2 RT-PCR negative and positive.^10^ However, this does not account for the proportion of patients who are SARS-CoV-2^neg^ but are considered to have COVID-19 clinically. The aim of this study was to assess if any routine COVID-19 blood tests^7^ could improve diagnostic accuracy in conjunction with that of the detection of SARS-CoV-2 RT-RCR following upper respiratory tract sampling. Specifically, we aimed to (1) identify how blood tests differ between patients who are ultimately diagnosed with COVID-19, and those who are not, in patients referred for possible COVID-19 and (2) attempt to mathematically combine blood test results together to assess if this could be used to support a diagnosis of COVID-19.

## Method

A retrospective review was performed on all patients consecutively referred to General Internal Medicine (GIM) over a period of four weeks at a UK NHS District General Hospital. Patients were only retrospectively included if the referral stated a clinical suspicion of COVID-19 and consequently a COVID-19 PCR-RT swab had been taken. These included Emergency Department (ED) referrals and direct general practitioner referrals. Patients who presented to the ED and who were sent home without admission were excluded. Patients with nosocomial transmission of SARS-CoV-2 were excluded unless the patient had been discharged and represented with symptoms of COVID-19.

The patient’s admission blood test results and radiographic results were noted as well as any other blood tests or scans performed within 48 h of admission (to account for missed-off tests or haemolysed samples and delays to initial diagnostic cross-sectional imaging). The presence of positive blood cultures was noted with coagulase-negative staphylococcal cultures excluded if considered (at the time) to be secondary to skin commensal contamination. Where duplicate tests were performed, the admission results only were included except for a chest computed tomography (CT) scan which was used preferentially to a chest radiograph (CXR). Radiology results (CXR^11^ and CT thorax^12^) were summated and categorized as being: ‘in keeping with COVID-19 related lung disease’ (e.g. for CXR: ground glass opacities, with bilateral, peripheral, and lower lung zone distributions), ‘not in keeping with COVID-19’ or ‘indeterminate’. Radiographic results were taken directly from the reporting radiologist/radiographer’s report with no primary interpretation performed. Reports were specifically written as being supportive, not supportive, or indeterminate for a diagnosis of COVID-19. Other presenting symptoms were also recorded. Symptoms were noted as per Wang et al.^5^ namely: fever, fatigue, dry cough, anorexia, myalgia, dyspnoea. Other presenting symptoms were recorded and categorized.

The patient’s length of inpatient admission and outcome were noted. The patients were divided on account of their discharge diagnosis into COVID or CONTROL. The COVID group were thought to have a clinical diagnosis of COVID-19 which considered the symptoms, signs, radiology and SARS-CoV-2 RT PCR swab (Kingfisher flex nucleic acid extraction system and ABI7500 Fast PCR machine; Thermo Fisher, Waltham, Massachusetts, USA). The CONTROL group were considered not to have COVID-19 using the same criteria. The groups were split based on the discharge diagnosis, using the clinical notes or discharge summary alone, with no retrospective interpretation. The COVID and CONTROL groups were compared. We also performed a subgroup analysis of the COVID group based on the SARS-CoV-2 RT-PCR result as detected (COVID^pos^RT-PCR^pos^) or not detected (COVID^pos^RT-PCR^neg^). Finally, we compared the COVID^pos^RT-PCR^neg^ group with the CONTROL group.

Due to the retrospective design, using anonymized data, no ethical approval was necessary for this service evaluation. Data were assessed for normality using the Shapiro-Wilk test prior to data analysis. Nominal data were compared using Fisher’s exact test and expressed as percentages. Parametric continuous measures were compared with Student’s *t*-test and expressed as mean and standard deviation. A Sensitivity Index (*SI*), as another measure of how much the distributions separated, was used to compare COVID and CONTROL blood tests. The Sensitivity Index is defined as (equation (1)):

(1) $$SI=\frac{\mu_{\mathrm{COVID}}-\mu_{\mathrm{CONTROL}}}{\sqrt{\frac{1}{2}(\sigma_{\mathrm{COVID}}^{2}+\sigma_{\mathrm{CONTROL}}^{2})}}$$

where μ and σ^2^ denote the mean and variance, respectively, of a distribution. A sensitivity threshold of >0.35 was used. Receiver operator curves (ROC) were performed on blood tests with significant (*P* < 0.05) differences between groups. The sensitivity and specificities were examined and the area under the curve (AUC) recorded. The α level was set to 0.05. Parametric and non-parametric analyses were performed using GraphPad Prism 8.0, GraphPad Software, San Diego, California. Sensitivity and specificity analysis were performed using Matlab, Mathworks, Natick, Massachusetts. A combined blood test was formulated and the AUC calculated. This was validated on a further retrospective dataset of 181 patients.

## Results

A total of 399 patients (cohort 1), 229 male (57%) and 170 female (43%), were referred to the GIM team for consideration of hospitalization, with suspected COVID-19, over a four-week period from 11 March 2020 to 8 April 2020. A clinical diagnosis of COVID-19 was made in 213 patients (53%) (COVID group) following consideration of symptoms, radiology, blood tests and a SARS-CoV-2 RT-PCR with 186 (47%) diagnosed with not having COVID-19 based on the same considerations (CONTROL group). A comparison of demographic details, in hospital mortality, symptoms and length of admission can be reviewed in Table 1. If less than 5% of patients had a specific symptom recorded, it was classified as ‘other’. The CONTROL group were significantly more likely to have ‘other’ symptoms (*P* < 0.0001) which included dizziness, sore throat, diarrhoea and/or vomiting, collapse, anorexia, palpitations, urinary retention, constipation, wheeze, hyperglycaemia, difficulty coping at home, headache, photophobia and rashes.

**Table 1.** A comparison of demographics and symptoms for all patients referred to General Internal Medicine with a potential diagnosis of COVID-19 who were ultimately diagnosed with COVID-19 (COVID) or another diagnosis (CONTROL).

		COVID (SD)	CONTROL (SD)	*P* value
Demographics
Male	(%)	62	53	
Female	(%)	38	47	0.069
Age	(years)	64.7 (18.1)	68.9 (19.5)	0.030
Death during admission	(%)	20.1	5.9	<0.0001****
Length of admission	(days)	5.8 (5.2)	4.8 (3.5)	0.059
Presenting symptoms
Cough	(%)	55	40	<0.0001****
Fever	(%)	51	35	0.0025**
Dyspnoea	(%)	46	51	0.302
Fatigue	(%)	9	4	0.090
Chest pain	(%)	8	14	0.068
Myalgia	(%)	6	6	>0.999
Confusion	(%)	5	6	0.645
Falls	(%)	4	5	0.46
Other	(%)	9	45	<0.0001****

Radiological (predominantly CXR) investigation was significantly different in COVID vs. CONTROL groups (*P* < 0.0001). Overall, 32% of patients in the COVID group did not have radiology in keeping with COVID-19, 18% were indeterminate, with 50% in keeping with changes typical of COVID-19 lung disease. By comparison 68% of patients in the CONTROL group had radiological findings not in keeping with COVID-19, 19% were indeterminate and 12% were in keeping with COVID-19.

### Blood tests

A comparison of blood tests between COVID and CONTROL can be reviewed in Table 2. In the COVID group, 49/213 patients (23%) had an influenza A, influenza B and respiratory syncytial viral swab; 78/186 patients (42%) in the CONTROL group. All swabs were negative except for one positive swab for influenza A in the CONTROL group. The sensitivity and specificity, in all patients, of the SARS-CoV-2 RT-PCR was 74.2% and 99.5%, respectively, in diagnosing COVID vs. CONTROL.

**Table 2.** A comparison of blood test results for all patients referred to General Internal Medicine with a potential diagnosis of COVID-19 who were ultimately diagnosed with COVID-19 (COVID) or another diagnosis (CONTROL).

		COVID (SD)	CONTROL (SD)	*P* value	Sensitivity Index	AUC
Haemoglobin	(g/l)	129 (23)	133 (89)	0.582		
White cell count	(10^9^/L)	8.1 (4.8)	11.6 (6.5)	<0.0001****	−0.61	0.69
Platelets	(10^12^/L)	229 (105)	265 (99)	0.0008***	−0.34	0.61
Neutrophils	(10^9^/L)	6.4 (4.6)	9.1 (5.7)	<0.0001****	−0.51	0.68
Lymphocytes	(10^9^/L)	0.9 (0.7)	1.6 (2.4)	0.0001***	−0.38	0.62
Urea	(mmol/L)	8.9 (11.3)	7.9 (5.5)	0.279		
Creatinine	(µmol/L)	96 (86)	85 (62)	0.136		
C-reactive protein	(mg/L)	96 (82)	84 (102)	0.188		
Albumin	(g/L)	40 (6.3)	41 (5.4)	0.336		
Alkaline Phosphatase	(U/L)	98 (83)	117 (120)	0.082		
Alanine transaminase	(U/L)	49 (52)	51 (142)	0.860		
Bilirubin	(µmol/L)	12 (10)	15 (16)	0.073		
Creatinine kinase	(U/L)	294 (538)	197 (449)	0.131		
High Sensitivity Troponin I	(ng/L)	273.8 (2150)	284.3 (2282)	0.968		
D-dimer	(ug/L)	2833 (9722)	2378 (3913)	0.686		
Aspartate aminotransferase	(U/L)	63 (58)	54 (61)	0.407		
Lactate dehydrogenase	(U/L)	620 (269)	475 (232)	<0.0001****	0.58	0.68
Ferritin	(µg/L)	937(1015)	384 (628)	<0.0001****	0.66	0.74
SARS-CoV-2 RT-PCR	(detected, %)	76	0.5	<0.0001****		0.89
Positive blood culture	(positive, %)	4	17	0.001**		

AUC: area under the curve; SD: standard deviation; SARS-CoV-2 RT-PCR: severe acute respiratory syndrome coronavirus 2 reverse-transcription polymerase chain reaction.

### COVID-19 patients with a negative SARS-CoV-2 RT-PCR

A subgroup analysis was performed to further elucidate the patients who were COVID^pos^RT-PCR^neg^. Overall, there were 51/213 (24%) patients who were COVID^pos^RT-PCR^neg^ but considered to have COVID representing 13% (51/399) of all patients referred with the suspicion of COVID-19 over the four-week collecting period. In this group, 23/51 (45%) patients had radiological findings suggestive of COVID-19, 5/51 (10%) had radiological findings which were indeterminate for COVID-19 and 22/51 (43%) had radiological findings not suggestive of COVID-19. In one patient, no radiological investigation was taken. Overall, 28/51 (55%) had a new persistent cough and/or fever. In three patients, symptoms were not recorded in the referral database. Overall, 35/51 (67%) patients had at least one of new persistent cough, new fever or chest X-ray changes in keeping with COVID-19 with 12/51 (24%) patients having neither. In four patients, the admission data were incomplete.

In comparing COVID^pos^RT-PCR^neg^ patients with the CONTROL patients, there was a significant difference in FER levels (CONTROL; 384 ± 628 µg/L, COVID^pos^RT-PCR^neg^; 985 ± 1344 µg/L, *P* = 0.001). COVID^pos^RT-PCR^neg^ patients were significantly more likely to have a radiological investigation in keeping with COVID in comparison to CONTROL (COVID^pos^RT-PCR^neg^ 45%, CONTROL; 12%, *P* < 0.0001). There were no significant differences in lymphocytes (LYM) (CONTROL 1.59 ± 2.39 10^9^/L, COVID^pos^RT-PCR^neg^ 1.11 ± 1.16 10^9^/L, *P* = 0.167) or white cell count (WCC) (CONTROL; 11.6 ± 6.5 10^9^/L, COVID^pos^RT-PCR^neg^; 11.2 ± 6.6 10^9^/L, *P* = 0.714) or any other blood test (*P* > 0.075). Death during admission was significantly higher (*P* < 0.0001) in the COVID^pos^RT-PCR^neg^ group (*n* = 15/51, 29%) compared to CONTROL (*n* = 11/186, 6%) despite the COVID^pos^RT-PCR^neg^ group being significantly younger (COVID^pos^RT-PCR^neg^; 61.8 ± 20.4 years, CONTROL; 68.9 ± 19.4 years) (*P* = 0.027).

In comparing COVID^pos^RT-PCR^neg^ patients with COVID^pos^RT-PCR^pos^ patients, there were no significant differences in length of stay (COVID^pos^RT-PCR^neg^; 5.0 ± 5.0, COVID^pos^RT-PCR^pos^ 6.1 ± 5.3, *P* = 0.309), sex (62% male in both groups), age (COVID^pos^RT-PCR^neg^; 61.8 ± 20.7, COVID^pos^RT-PCR^pos^; 65.3 ± 17.1, *P* = 0.219), death during admission (COVID^pos^RT-PCR^neg^; 29.4%, COVID^pos^RT-PCR^pos^; 17.3%, *P* = 0.072), any symptom (*P* >0.097) or radiological manifestations of COVID-19 (COVID^pos^RT-PCR^neg^; 49.7%, COVID^pos^RT-PCR^pos^; 45.1%, *P* = 0.628). However, there were significantly raised WCC (COVID^pos^RT-PCR^neg^; 11.2 ± 6.6 × 10^9^/L, COVID^pos^RT-PCR^pos^; 7.1 ± 3.6 × 10^9^/L, *P* < 0.0001), neutrophils (NEU) (COVID^pos^RT-PCR^neg^; 9.15 ± 6.5 × 10^9^/L, COVID^pos^RT-PCR^pos^; 5.6 ± 3.4 × 10^9^/L, *P* <0.0001), platelets (PLTS) (COVID^pos^RT-PCR^neg^; 265 ± 120 × 10^12^/L, COVID^pos^RT-PCR^pos^; 219 ± 98 × 10^12^/L, *P* = 0.0063) and LYM (COVID^pos^RT-PCR^neg^; 1.1 ± 1.2 × 10^9^/L, COVID^pos^RT-PCR^pos^; 0.9 ± 0.4 × 10^9^/L, *P* = 0.025) in comparison to the COVID^pos^RT-PCR^pos^. Alkaline phosphatase (ALP) (COVID^pos^RT-PCR^neg^; 145 ± 138 U/L, COVID^pos^RT-PCR^pos^; 83 ± 48 U/L, *P* < 0.0001), Bilirubin (COVID^pos^RT-PCR^neg^; 16 ± 13µmol/L, COVID^pos^RT-PCR^pos^; 11 ± 8 µmol/L, *P* = 0.038) and High Sensitivity Troponin I (COVID^pos^RT-PCR^neg^; 1058 ± 4536ng/L, COVID^pos^RT-PCRpos; 52 ± 131ng/L, *P* = 0.012) were also significantly increased in the COVID^pos^RT-PCR^neg^ patients. There was no significant difference in FER (COVID^pos^RT-PCR^neg^; 985 ± 1344µg/L, COVID^pos^RT-PCR^pos^; 925 ± 918µg/L, *P* = 0.768), CRP (*P* = 0.690) or renal function (Creatinine, estimated glomerular filtration rate and Urea; *P* > 0.315) as well as Albumin (*P* = 0.268), alanine transaminase (ALT) (*P* = 0.213), aspartate aminotransferase (AST) (*P* = 0.108), d-dimer (*P* = 0.595) and creatine kinase (*P* = 0.185). The LDH (COVID^pos^RT-PCR^neg^ 515 ± 176 U/L, COVID^pos^RT-PCR^pos^ 643 ± 283 U/L, *P* = 0.312) and haemoglobin (COVID^pos^RT-PCR^neg^; 121 ± 28 g/L, COVID^pos^RT-PCR^pos^; 132 ± 21 g/L, *P* = 0.0042) were significantly lower in the COVID^pos^RT-PCR^neg^ patients.

### The COVID Combined Blood Test (CCBT)

The COVID and CONTROL groups were compared to attempt to produce a combined blood test. Only complete records were used. In comparing COVID and CONTROL, five blood tests (WCC, NEU, LYM, LDH and FER) had a sensitivity index >0.35 and the means were significantly different to reject the null hypothesis (α = 0.05) (Table 2).

The output numerical values of the five blood tests for each patient were linearly combined with weights that maximized the AUC. The weights were estimated using a non-parametric stepwise method^13^ according to the following procedure:

1. Estimate the AUC from the output values for each of the five individual blood tests;
2. Order the blood tests according to their estimated AUC from largest to smallest;
3. Estimate by a search routine the proportion of the output value of the blood test with the second largest AUC that when added to the output value of the blood test with the largest AUC produces a maximum combined AUC;
4. Proceed in this fashion until the blood test with smallest AUC is included in the linear combination.

The resulting combination is called the COVID CCBT score. NEU and LDH were found to make negligible contribution to the weighted combination. Mathematically, the definition of the CCBT then reduced to (equation (2)):

(2) $$\mathrm{CCBT}=\frac{\mathrm{FER}}{705.2}-\frac{\mathrm{WCC}}{8.473}-\frac{\mathrm{LYM}}{11.59}$$

where, for example, WCC denotes the numerical value of a WCC blood test. The negative sign for WCC and LYM accounts for their negative sensitivity.

**Figure 1.**
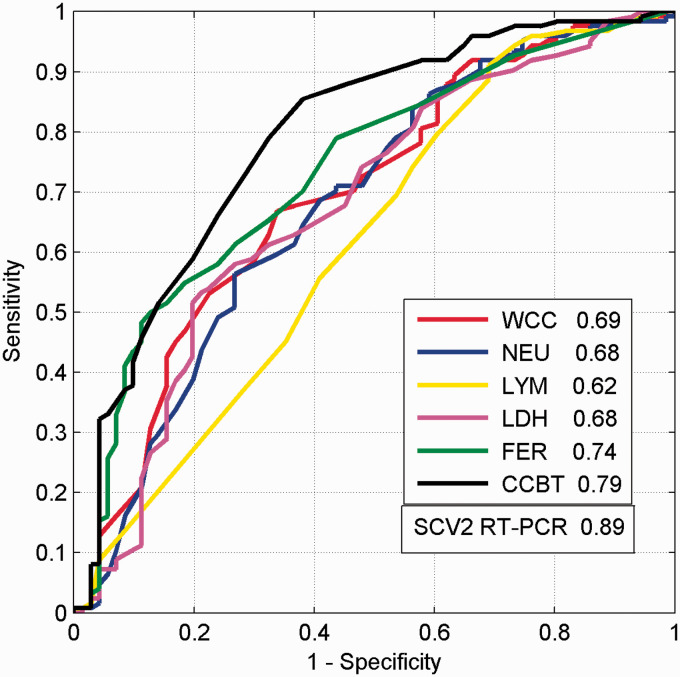
Receiver operator curves (ROC) and area under the curve (AUC) for the CCBT (COVID Combined Blood Test) and the individual ROCs and AUCs for WCC (white cell count), NEU (neutrophils), LYM (lymphocytes), LDH (lactate dehydrogenase) and FER (ferritin), with SCV2 RT-PCR (severe acute respiratory syndrome coronavirus 2 reverse-transcription polymerase chain reaction) included for comparison.

**Figure 2.**
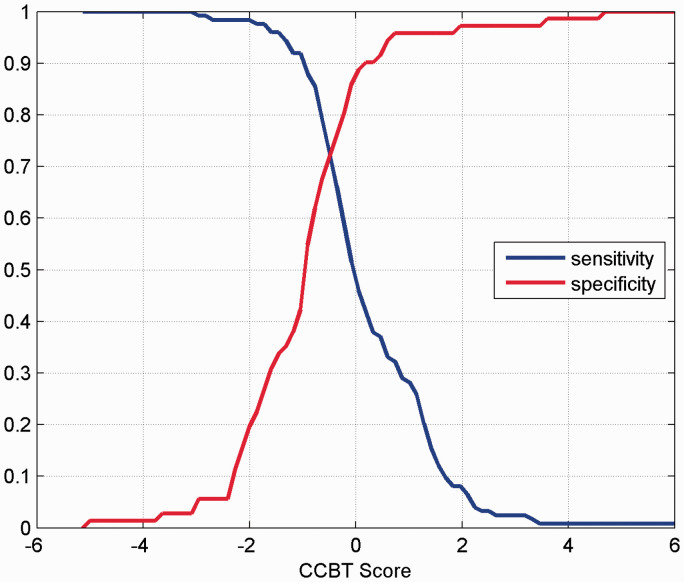
The sensitivity and specificity as a function of the output of the COVID Combined Blood Test (equation (2)) for the white cell count, lymphocytes and ferritin data sets.

The ROC and associated AUC were calculated for this CCBT, together with the individual ROCs and AUCs for the constituent blood tests WCC, NEU, LYM, LDH and FER (Figure 1). The AUC for this CCBT was 0.79. Figure 2 shows a plot of the sensitivity and specificity as a function of the output of the CCBT. For a given patient, the numerical readings for the three blood tests FER, WCC and LYM are substituted into equation (2) to provide a CCBT score. With reference to Figure 2, a threshold CCBT value is chosen corresponding to the selected pair of sensitivity and specificity values. The decision COVID/non-COVID is made according to whether the calculated CCBT score lies above or below the threshold. We have used a CCBT threshold of –0.8 to exemplify this in terms of sensitivity and specificity, where <–0.8 was CCBT negative and ≥–0.8 was CCBT positive (Table 3).

**Table 3.** The sensitivity, specificity of the CCBT (equation (2)), in comparison to the SARS-CoV-2 RT-PCR on a validating cohort of 181 patients in comparison of 399 patients.

		COVID-19 diagnosis					
Dataset	Test	True positives	True negatives	False positives	False negatives	Sensitivity	Specificity
Initial data (*n* = 399)	CCBT	127	57	34	23	0.85	0.63
	SARS-CoV-2 RNA	122	91	0	28	0.81	1.00
Validation data (*n* = 181)	CCBT	64	44	15	24	0.73	0.75
	SARS-CoV-2 RNA	61	59	0	27	0.69	1.00

Blood test data were retrospectively collected from a further 181 patients similarly suspected of COVID-19, over a further two-week period (9 April to the 24 April inclusive). Overall, 56% (101/181) were confirmed as COVID-19 (male; 51.5% age; 71.5 ± 19.4) on discharge diagnosis of which 32 patients (32%, 32/101) were COVID^pos^RT-PCR^neg^. In comparing this dataset with the initial 399 patients, in the COVID groups, the patients were significantly older in the later group (*P* = 0.0027) but there was no significant difference in age (*P* = 0.0843) or mortality (*P* = 0.137). There was no significant difference in individual blood test results in comparing full datasets, COVID or CONTROL groups. The CCBT was tested on this second dataset resulting in an AUC of 0.78, providing some evidence of its validation. Sensitivity and specificity can be seen in Table 3.

## Discussion

While there has been an uplift of testing capability for COVID-19, intermittent delays to hospital diagnosis remain, particularly in low-resource settings or in the instance of the patient being COVID^pos^RT-PCR^neg^. While rapid, accurate testing capabilities continue to evolve, access to these are variable, and accuracy has only been evaluated in small numbers.^14^ As hospitals address the backlog of elective work, coupled with addressing the non-COVID emergency work, there will still be the requirement to accurately and rapidly diagnose COVID-19 patients and quickly transition patients from ED. Many hospitals, such as ours, are using a separate ward or facility, for potential COVID-19 patients to curb the nosocomial transmission among inpatients, many of whom would be highly susceptible to severe manifestations of COVID-19.

We found a significantly lower WCC, NEU, PLTS and LYM in COVID-19 patients and a significantly higher LDH and FER in comparison to patients who were not ultimately diagnosed with COVID-19 despite a clinical suspicion. This may assist in early discrimination of patients who are more likely to have COVID so allowing for more accurate quarantine. Our study used a clinical endpoint for COVID-19 diagnosis so attempting to capture the 15–20% of patients who are thought to have COVID-19 but are COVID^pos^RT-PCR^neg^. Using these results, in conjunction with a sensitivity index, we have been able to mathematically combine the WCC, LYM and FER blood test results to attempt to improve clinical acumen in diagnosing COVID-19. We have named this the COVID Combined Blood Test. This score has the potential to improve clinical acumen in diagnosing COVID-19 while awaiting formal diagnosis (Figure 2). Validation shows using a CCBT score of –0.8 results in a sensitivity of 0.75 and a specificity of 0.73. While this is insufficient to reliably make the diagnosis alone, the sensitivity is comparable to a SARS-CoV-2 RT-PCR swab. This could be used in conjunction with the clinical presentation and radiological investigations to assist the clinician in patient diagnosis and flow particularly in low-resource settings, where the SARS-CoV-2 PCR is suspected to be falsely negative, or where delays exist.

The addition of radiology and symptoms may improve the score’s sensitivity and specificity further although these require a degree of interpretation and so were not utilized on this first iteration. In retrospect this was the correct course of action as subsequently there was the recognition of anosmia and ageusia as prominent COVID-19 symptoms^15^ as well as the variable use of CT scanning to improve sensitivity.^3^ The CCBT may require further refinement and prospective validation to perform optimally although remained robust on a further retrospective validating dataset. We opted not to retrospectively alter the clinical diagnosis based on further interpretation of the patient’s clinical presentation. While this may have improved confidence in the integrity of the COVID and CONTROL groups, we felt it had equal potential to introduce bias.

Studies have assessed blood testing for the purposes of diagnosis in COVID-19. A study of 207 patients admitted with symptoms of coronavirus compared blood tests between those who tested negative and those testing positive on SARS-CoV-2 RT-PCR. They did not record symptoms and viral RNA by RT-PCR was the gold standard. WCCs, NEU and LYM were significantly lower with AST, ALT and LDH significantly higher.^16^ Here they attempted to use LDH and AST to positively and negatively predict COVID-19. FER was not recorded. It is interesting that these largely reflect our results other than for AST. We also found PLTS and NEU to be significant which may be due to our larger dataset. A further Iranian prospective study by Mardani et al.^10^ of 200 cases assessed the accuracy of routine blood tests only to evaluate the accuracy of laboratory parameters in predicting cases with COVID-19 positive RT PCR. WCC, LYM and albumin were significantly lower in RT-PCR positive patients with NEU, CRP, AST (aspartate transaminase), ALT, LDH and Urea significantly higher. CRP, ALT, LDH, Urea and NEU all had an AUC of >0.8. We were unable to reproduce this and one can only presume a differing patient population.

This retrospective study has several limitations. These data are built on secondary care patients who were referred to the GIM team with a presentation consistent with COVID-19 which serves as a loosely defined pre-test probability for the CCBT. These criteria could however be more defined to assist with the varying incidence of COVID-19 over time. That we did not compare blood tests to a more reproducible and predictable end point such as a positive SARS-CoV-2 RT PCR result undoubtedly introduced bias. As we did not retrospectively interpret the clinical diagnosis, and with the lack of a gold standard for testing, we cannot be absolutely confident that all patients in the COVID group had COVID-19 and that all patients in the CONTROL group did not have COVID-19. However, we wanted to try and capture this diagnostically challenging group of RT-PCR negative COVID-19 patients. We considered that patients who were COVID^pos^RT-PCR^neg^ would likely only be diagnosed as such based on a typical presentation and radiological findings. While this was the case in 68% of patients, these initial diagnostic data were only captured peri-admission and therefore did not account for repeated SARS-CoV-2 testing, radiological investigations performed after 48 h, close contact with known SARS-CoV-2-infected persons, further collateral history, or symptoms such as anosmia which, at that time, had not been absolutely determined. While these data would have been used to form the discharge diagnosis it did not inform thinking peri-admission. Furthermore, while we made every effort to exclude nosocomial spread, this also cannot be excluded absolutely in this group particularly in patients with repeat positive PCR diagnosis 48 h after admission. We could have improved the credibility of a COVID-19 diagnosis in this important group with an independent panel of clinicians retrospectively judged the veracity of the discharge diagnoses.

The significant differences in blood test results for COVID^pos^RT-PCR^neg^ and COVID^pos^RT-PCR^pos^ can potentially be explained by a false-positive diagnosis of the COVID^pos^RT-PCR^neg^ patients as having COVID-19. It is possible however that the patients who were thought to have COVID-19 but were COVID^pos^RT-PCR^neg^ merely presented later, so had cleared the virus from their nasopharyngeal tract, but were temporally more likely to be suffering COVID-19 related complications in keeping with the relative leucocytosis, neutrophilia, thrombocytosis, liver function tests and troponin in the COVID^pos^RT-PCR^neg^ group. That we did not account for when the patients became symptomatic is a further limitation. These data suggest that in COVID^pos^RT-PCR^neg^ patients FER is the most useful discriminating blood test. FER must however be interpreted in the context of sex, iron status and pre-morbid disease (particular liver disease) but can also be raised in other inflammatory and infective conditions as an acute phase protein. Finally, it is equally plausible that there was an anchor bias in the CONTROL group to a negative SARS-CoV-2-RT-PCR result leading to a false-negative diagnosis and so obscuring the comparison between COVID and CONTROL. For instance, 12% of the CONTROL group had radiological findings in keeping with COVID-19 but were ultimately not thought to have had COVID-19. This potentially could have been further granulated if we collected the final discharge diagnosis of the CONTROL group rather than just ascertaining that COVID-19 was not suspected as the cause of the patient’s presentation. We would consider that while false positives may be an issue in asymptomatic testing, in the context of symptomatic hospital admissions COVID^neg^RT-PCR^pos^ is less likely accounting for the 100% specificity of this.

This study also does not address asymptomatic COVID-19 patients who may not have similar blood test pictures as those who were hospitalized, as in this study. This study collected data between the 11 March and 8 April which saw a rising expansion in COVID-19 cases requiring rapid reorganization of care in NHS hospitals. As the prevalence increased, the clinician’s suspicion of COVID-19 is also likely to have increased, which coupled with enhanced capacity for testing will have resulted in an inconsistent approach to testing for SARS-CoV-2 RT-PCR over time with nearly all patients suspected as having COVID towards the end of our designated collection period. This introduces significant bias regarding symptoms. While we saw significant differences between COVID and CONTROL, the clinical suspicion may have been greater in the COVID group leading to more testing of COVID-19 parameters (e.g., FER, LDH, d-dimers) so confounding the results.

## Conclusion

Patients presenting with potential diagnosis of COVID-19 require rapid accurate diagnosis to halt viral transmission. The diagnosis of COVID-19 can be challenging due to false negatives and delays in the processing of SARS-CoV-2 RNA RT-PCR on nasopharyngeal swab. There are significant differences in WCC, NEU, PLTS, LYM, LDH and FER in COVID-19 patients in comparison to patients where there is a clinical suspicion, but who are ultimately not diagnosed with COVID-19. These can be used, in conjunction with radiology and symptoms, to assist in the diagnosis. This is of particular use in a low-resource setting or where there are delays to definitive diagnosis. FER appears to be the most reliable blood marker and may be of particular use where there remains a clinical suspicion of COVID-19 despite a negative SARS-CoV-2 RT-PCR. It is possible to combine standard blood test results (WCC, LYM and FER) with a sensitivity comparable to SARS-CoV-2 RT-PCR, with the potential to assist in diagnosing COVID-19, and we have described one simple method, but this requires further prospective validation.
